# Regarding data visualization

**DOI:** 10.1017/ice.2020.457

**Published:** 2021-09

**Authors:** David Birnbaum

**Affiliations:** Applied Epidemiology, Sidney British Columbia, School of Population & Public Health, University of British Columbia, Vancouver, British Columbia, Canada; School of Health Information Science, University of Victoria, Victoria, British Columbia, Canada

*To the Editor—*The review by Salinas et al^1^ introduces many important aspects concerning the science of data visualization. However, the references cited in support of an assertion that the best ways to visualize data remain unclear overlooks several important resources that provide insightful practical advice on optimal choices. In particular, the work of William Cleveland, whose career was devoted to scientific study of visual encoding and decoding of scientific data, and the work of various cognitive psychologists are noteworthy. Cleveland’s findings are distilled into 2 very useful books that have been reviewed in this journal.^2,3^ Important findings from cognitive psychology articles are distilled into various comprehensive review publications, like that of Gigerenzer et al.^4^ The graph examples illustrated by Salinas et al should be viewed with key concepts from Cleveland and Gigerenzer in mind. Exploratory data analysis methodology based on data visualization principles and techniques established in the 1970s–1990s “… add an exciting and useful tool to the epidemiologist’s repertoire.”^5^ The works of Cleveland, Gigerenzer, and others were paramount in informing many of the choices I had to make (and defend against those who initially found them unfamiliar) throughout my career in hospital and public health agency projects related to recognizing the onset of adverse trends efficiently and informing a wide range of audiences about comparisons of healthcare-associated infection rates.^6–9^

## References

[1] Salinas JL, Kritzman J, Kobayashi T, et al. A primer on data visualization in infection prevention and antimicrobial stewardship. Infect Control Hosp Epidemiol 2020;41:948–957. 32389138 10.1017/ice.2020.142

[2] Birnbaum D. Book review, re: Cleveland WS, *Visualizing Data* . Infect Control Hosp Epidemol 1994;15:763.

[3] Birnbaum D. Book review, re: Cleveland WS, *The Elements of Graphing Data* . Infect Control Hosp Epidemiol 1996;17:706.

[4] Gigerenzer G, Gaissmaier W, Kurz-Milcke E, Schwartz LM, Woloshin S. Helping doctors and patients make sense of health statistics. Psychol Sci Public Interest 2007;8:53–96. 26161749 10.1111/j.1539-6053.2008.00033.x

[5] Shelly MA. Exploratory data analysis: data visualization or torture? Infect Control Hosp Epidemiol 1996;17:605–612. 8880234 10.1086/647397

[6] Birnbaum D, Cummings MJ, Guyton K, Schlotter J, Kushniruk A. Designing public web information systems with quality in mind: public reporting of hospital performance data. Clin Govern Int J 2010;15:272–278.

[7] Hyman DA, Black BS. Public reporting of hospital infection rates: not all change is progress (January 2013). Jurimetrics. Forthcoming, Northwestern Law & Econ Research Paper No. 12-21, Northwestern University, Institute for Policy Research Working Paper 13-07, Illinois Program in Law, Behavior and Social Science Paper No. LE13-18. Social Science Research Network website. https://ssrn.com/abstract=2219510. Published February 2013. Accessed September 12, 2020.

[8] Birnbaum D.Chapter 4, Big data challenges from a public health informatics perspective. In Househ M, Borycki E, Kushniruk A, eds. Big Data, Big Challenges: A Healthcare Perspective. New York: Springer International, 2019.

[9] Birnbaum D.Chapter 9, Epidemiologic methods for investigating infections in the healthcare setting. In Jarvis WR, ed. Bennett & Brachman’s Hospital Infections, 7^th^ edition. Philadelphia: Wolters Kluwer; in press.

